# Physiotherapeutic Protocol and ZnO Nanoparticles: A Combined Novel Treatment Program against Bacterial Pyomyositis

**DOI:** 10.3390/biology11101393

**Published:** 2022-09-23

**Authors:** Hesham El-Shaer, Bassma H. Elwakil, Basant A. Bakr, Ahmed M. Eldrieny, Mostafa El-Khatib, Khim Phin Chong, Amr A. Abo Gazia

**Affiliations:** 1 Faculty of Physical Therapy, Pharos University in Alexandria, Alexandria 21500, Egypt; 2 Faculty of Applied Health Sciences Technology, Pharos University in Alexandria, Alexandria 21500, Egypt; 3 Faculty of Science, Alexandria University, Alexandria 21544, Egypt; 4 Faculty of Engineering, Pharos University in Alexandria, Alexandria 21500, Egypt; 5 Faculty of Science and Natural Resources, Universiti Malaysia Sabah, Jalan UMS, Kota Kinabalu 88400, Sabah, Malaysia; 6 Faculty of Physical Therapy, Kafr Elsheikh University, Kafr Elsheikh 33516, Egypt

**Keywords:** pyomyositis, infected rat model, physiotherapeutic program, zinc oxide nanoparticles

## Abstract

**Simple Summary:**

Bacterial infections are among the widely disturbing diseases and leading cause of mortality around the world. Some bacterial strains can invade the skeletal muscles by producing proteolytic enzymes, causing pyomyositis. The aim of the present investigation was to evaluate ZnO nanoparticles (ZnO-NPs) as an effective treatment for bacterial pyomyositis in combination with a physiotherapeutic program (consisting of swimming and a low-level laser treatment (LLLT)). Results revealed the significant and synergistic effect of both ZnO-NPs along with the physiotherapeutic program in enhancing the histoarchitecture, immunity and hemostasis, and some vital physiological biomarkers. This emphasizes the urgency of spreading more awareness about the importance of the combined effect between nanotechnology and physiotherapeutic programs in muscle regeneration after pyomyositis caused by virulent infection with proteolytic bacteria.

**Abstract:**

*Myositis tropicans* or pyomyositis is a muscle inflammation resulting from a bacterial infection of skeletal muscle (commonly caused by *Staphylococcus aureus*) that usually leads to hematogenous muscle seeding. The present study was designed to estimate the role of ZnO-NPs and a physiotherapeutic program in the management of induced *biceps femoris* atrophy in rats through histological, biochemical, and radiological examinations at different time intervals. At the beginning, several bacterial strains were evaluated through a proteolytic enzyme activity assay and the highest activity was recorded with the *Staphylococcus aureus* strain. ZnO-NPs were synthesized with the arc discharge method with an average size of 19.4 nm. The antibacterial activity of ZnO-NPs was investigated and it was revealed that the prepared ZnO-NPs showed a minimum inhibitory concentration of 8 µg/mL against the tested bacterium. The cytotoxicity of the prepared ZnO-NPs was tested in C2C12 myoblast cells, and it was elaborated that CC50 was 344.16 µg/mL. *Biceps femoris* pyomyositis was induced with a potent strain (*Staphylococcus aureus*); then, a physiotherapeutic program combined with the prepared ZnO-NPs treatment protocol was applied and evaluated. The combined program claimed antibacterial properties, preventing muscle atrophy, and resulted in the most comparable value of muscle mass.

## 1. Introduction

Muscle atrophy is a frequently complicated ailment in the clinical population, exacerbated by various considerable functional consequences, despite the fact that there are multiple unique pathophysiological triggers [1]. Atrophy can be defined as the observed decrease in tissue or organ size, consequently leading to cellular shrinkage caused by the organelle’s loss of cytoplasm and proteins [2]. During atrophy, both myofibrillar and soluble protein degradation increase [3]. This atrophy interferes with people’s daily tasks, such as climbing stairs and even walking, leading to a loss of independence, a lower quality of life, and bad health [4]. Accordingly, sustaining skeletal muscle mass and function is crucial for mobility, disease prevention, general health, and quality of life, particularly since the skeletal muscles not only serve as an important part of whole-body metabolic regulation, but they also account for 40–45 percent of the body’s mass [5]. Pyomyositis is an acute bacterial infection of skeletal muscles that ideally results in abscess formation, and its incidence has generally been observed in a total of 4% of the adult population after surgical admission. The pyomyositis mortality rate ranges from 1 to 23% with *Staphylococcus aureus,* as the most commonly isolated organism [6].

One main solution in the combat against infectious diseases is the use of nanosized inorganic metal oxides due to their high surface area to volume ratio, unique physicochemical properties, and the various outstanding biological activities. More recently, zinc oxide nanoparticles (ZnO-NPs) have attracted researcher attention due to their safety [7,8]. ZnO-NPs are used as a powerful wide-spectrum antibacterial agent (against Gram-positive and Gram-negative bacteria) with a cidal effect due to the generation of reactive oxygen species (ROS) along with the rupture of the bacterial cell membrane [8]. Moreover, ZnO-NPs proved their ability to enhance bone and soft tissue regeneration [8].

In another avenue, physiotherapy is a provisional therapy that is best utilized in conjunction with standard chemical medication, despite the fact that it is sometimes misunderstood as an alternative. Physiotherapy is a field of medicine that incorporates a variety of physical disciplines to help relieve injuries, restore mobility, and improve physical function [9]. In health recruitment, management, treatment, and rehabilitation, the advantages of physiotherapeutic intervention have been thoroughly applied in muscle atrophy studies [9,10]. Several techniques can be employed in physiotherapy programs, such as the low-level laser treatment (LLLT), which was proven to be efficient in decreasing the latency of acute inflammation and enhancing tissue regeneration in muscle and tendon lesions [11]. Swimming was utilized to enhance muscle growth by reducing intracellular protein breakdown via downregulating autophagy signals. Recent research explained that combining swimming and the low-level laser therapy program was the most potent recommended program in the management of atrophy [12].

This research aimed to examine the various therapeutic strategies for the management of induced pyomyositis in the *biceps femoris* by emphasizing physiotherapeutic therapy as a supplemental strategy with chemical medication.

## 2. Materials and Methods

### 2.1. Bacterial Sample Collection and Identification

Tissue biopsy samples were provided by the routine lab of the main university hospital, Alexandria, Egypt. Then, samples were inoculated on blood and MacConkey agar plates to isolate the proteolytic bacterial strains. Each isolated strain was identified with the RapID™ ONE System (ref: R8311006) and Vitek 2 automated system (bio Merieux, Marcy l’Etoile, France). The spot inoculation technique on collagen and casein agar plates was used to screen the proteolytic activity, and the hydrolysis zone was measured using Coomassie brilliant blue (CBB) dye. Then, the proteolytic enzyme activity of the potent strain was measured according to the Folin–Lowry method [13] at 415 nm at different time intervals (3–12–20–24 h), incubated with azocasein as the substrate.

### 2.2. ZnO Nanoparticle Preparation

ZnO nanoparticles were synthesized with the arc discharge method following the method of El-Khatib et al. [14]; in the current work, the developed system was built locally and utilized to prepare ZnO. The electrodes had 2 mm width, 5 cm length, and 50 mm distance between them. The power source was selected to be a DC power supply in order to achieve a high yield with an operating voltage of 110 V and a current of 30 A. Chemical agents were not used in the procedure. The medium was a pure methanol solution. The formed nanostructures from zinc metal vapors were separated through three stages: nucleation, cluster growth, and condensation in dielectric media. To prevent the aggregation of nanoparticles, an ultracentrifuge model (Hettich MIKRO, Tuttlingen, German) was used for 10 min at 12,000 rpm; then, the yield was rapidly withdrawn from the media.

#### Physicochemical Characterization of Zinc Oxide Nanoparticles

The generated nanoparticles were investigated using conventional procedures. ZnO nanopowders (ZnO-NPs) were suspended in ethanol, sonicated, and then deposited onto a copper grid, before drying and studying with a JEOL-2100 HR-TEM. Powder X-ray diffraction (XRD, Philips, X’pert, Cu K) and zeta potential investigations of the produced ZnO-NPs were conducted. An energy-dispersive X-ray spectrometer (EDX) was also used to determine the elemental composition of the generated particles [15].

### 2.3. In Vitro Studies

#### 2.3.1. Antimicrobial Activity of the Synthesized ZnO-NPs

The antimicrobial activity of the synthesized ZnO-NPs was tested in vitro against the proteolytic enzyme-producing microorganism(s) through the disc-diffusion technique. Moreover, the antimicrobial activity was also evaluated by estimating the minimum inhibitory concentration (MIC) with the microdilution method [15]. All the tests were conducted in triplicate.

#### 2.3.2. Cytotoxicity of the Synthesized ZnO-NPs

The C2C12 mouse myoblast cell line was obtained from the American Type Culture Collection (ATCC via Sigma, Melbourne, Australia). The cells were grown on Dulbecco’s modified Eagle’s medium (DMEM, Gibco, New York, NY, USA) with 10% inactivated fetal calf serum and 50 µg/mL gentamycin. The myoblast cells were maintained with 5% CO_2_ at 37 °C in a humidified atmosphere and were subcultured three times/week. For cytotoxicity assays, the cell lines were suspended (5 × 10^4^ cell/well) and the ZnO-NPs were then added to 96-well plates (three replicates) to achieve eight different dilutions. After incubating for 24 h, the numbers of viable cells were counted and reported with the use of the MTT test. The survival curve was drawn (Graphpad Prism software (version 9.4.1, San Diego, CA, USA)) by assessing the relation between surviving cells and the nanoparticle concentrations. The cytotoxic concentration (CC_50_), which is the concentration required to inhibit 50% of intact cells, was estimated from the graphic plots of the survival curve [16].

### 2.4. In Vivo study

#### 2.4.1. Experimental Animals

Thirty-five healthy adult male Albino rats were obtained from the animal house of Faculty of Pharmacy and Drug Manufacturing, Pharos University in Alexandria, Egypt. The animal study was approved by the Animal Care and Use Committee (ACUC), Faculty of Science, Alexandria University (AU/04/21/01/28/9/02). Rats of 8 weeks old and weighing 180–200 g each were chosen when the experimentation commenced. The animals were housed (7 rats/cage) and all the rats were observed for health status and acclimatized under the controlled environmental conditions at room temperature (25 ± 2 °C), humidity (55 ± 5%), and at a normal day/night photoperiod (12 h dark and 12 h light cycle). Animals were allowed free access to food and drinking water ad libitum throughout the study.

**Experimental design:**

The experimental rats were divided into 5 different groups, and each group contained seven rats (*n* = 35). The experiment was conducted for one month.

**Group I, negative control group**: As a placebo, normal saline (0.9% NaCl, 1 mL) was injected intramuscularly (*biceps femoris*).

Animals of the four other groups received 1 mL intramuscular injections of *Staphylococcus aureus* suspension (5.27 × 10^8^ CFU/mL) to induce *biceps femoris* pyomyositis or muscle injury (atrophy) at the beginning of the experimental study; then, the animals were distributed as follows:

**Group II, positive control group**: induced *biceps femoris* pyomyositis; without treatment.

**Group III, experimental treated group CH (CHTG)**: induced *biceps femoris* pyomyositis and treated with 0.5 mL intraperitoneal injection of zinc oxide nanoparticles (50 mg).

**Group IV, experimental treated group PH (PHTG)**: induced *biceps femoris* pyomyositis and physiotherapeutic protocol was applied (low-level laser therapy (LLLT (AlGaInP; *λ* = 670 nm; 18 J/cm^2^ for 60 s; and 30 J/cm^2^ for 80 s; alternatively, 40 mW/cm^2^; 0.5 cm^2^ spot size)) and swimming (10 min)) three times per week [17].

**Group V, experimental treated group PH + CH (PCTG)**: induced *biceps femoris* pyomyositis and treated with a combined treatment regimen of ZnO-NPs (chemical) and the physiotherapeutic program.

#### 2.4.2. Histopathological Study

The histopathological examination of *biceps femoris* muscle sections taken from the hind limb at different time intervals (7th, 14th, 21st, and 28th day postinfection) was examined. At each time interval, one rat was randomly selected from each group, sacrificed, and had its *biceps femoris* removed, which was washed in normal saline, fixed, and dehydrated with ethanol (80–100%). Tissue samples were embedded in paraffin and sliced into 5 μm sections using a microtome, deparaffinized in xylene, then stained using H&E stains for histopathological examination using a light microscope.

#### 2.4.3. Blood Hematological and Biochemical Analyses

To collect blood samples, all rats were anesthetized with methoxyflurane (Pittman Moore, Inc., Washington Crossing, NJ, USA). After being sedated, approximately 3 mL of whole blood was taken via the dorsal aorta. Blood samples were collected in EDTA (for hematological analysis) and sterile tubes (for biochemical analysis) individually. After serum separation, liver functions, namely, SGOT, SGPT, total albumin, alkaline phosphatase, and creatine phosphate, were measured using an automated biochemical analyzer.

#### 2.4.4. Radiological Analysis

##### Computed Tomography (CT) Imaging Technique

All CT muscle studies were performed using a Siemens horizon CT scanner. The thigh and upper femur were examined through typical anatomical locations for skeletal CT muscle measurements (tube voltage of 80 kV, exposure 200 mAs, slice thickness of 1 mm). CT acquisition and reconstruction parameters were fixed across all the different study groups. All rats were imaged in a fixed prone position under anesthesia. Both right and left (*biceps femoris*) were countered for each rat, and a calculated volume was generated in cm^3^ after those muscle sizes were compared to determine the muscle recovery [18]. The added CT figures were analyzed with the treatment-planning system (TPS) Eclipse TM software (version 10.0, The Eclipse Foundation, Ottawa, ON, Canada). The delineation of the target muscle volume was described according to HU, where, thus, for a properly calibrated scanner, the CT values of the skeletal muscles were also termed muscle tissues, which were approximately 40 to 70 HU according to Schrauwen-Hinderling [19].

### 2.5. Statistical Analysis

ANOVA statistical comparisons performed with the F-test were used to assess the distinctions between the different treated groups and the control group. The IBM SPSS software program version 24.0 (IBM, Armonk, NY, USA) was used. The results of significance tests were expressed as two-tailed probability. The significance of the acquired results was determined at the 5% level. Moreover, principal component analysis (PCA) was performed to differentiate among different experimental rat groups using ORIGINPro^®^ 2022b (OriginLab, Inc., Northampton, MA, USA).

## 3. Results

### 3.1. Nanoparticles Characterization

**Figure 1:**
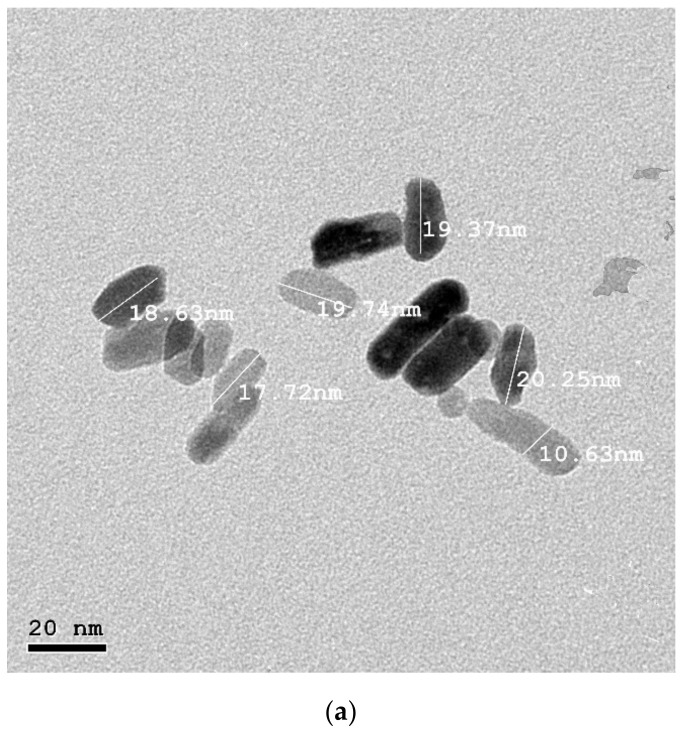

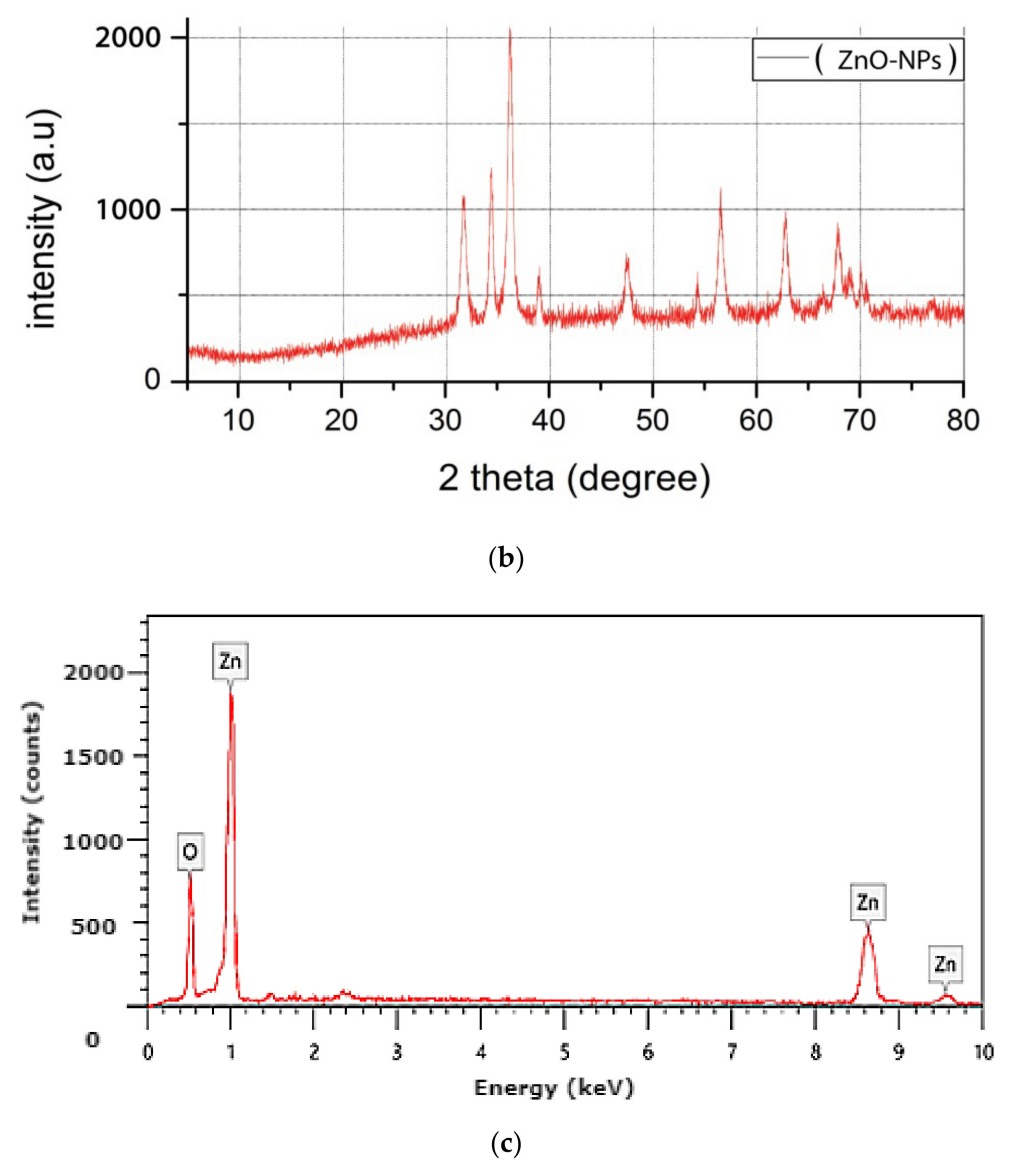
HR-TEM analysis (**a**), XRD analysis (**b**), and energy dispersive X-ray (EDX) (**c**) of the prepared ZnO-NPs.

HR-TEM was applied to investigate the morphology of ZnO-NPs. Figure 1a showed ZnO-NPs had a regular hexagonal structure with an average size of 19.4 nm. An XRD analysis was used to study the crystalline properties of the produced nanostructures which are depicted in Figure 1b. The appeared diffraction peaks at 2θ = 31°, 34°, 36°, 47°, 56°, 62°, 65°, 67°, 68°, and 76.4° corresponded to the (100), (002), (101), (102), (110), (103), (200), (112), (201), and (202) crystalline planes of the ZnO phase, respectively. All diffraction peaks were indexed by comparison with the data from a standard JCPDS card, no 36-1451. The chemical purity of the product was elucidated with energy dispersive X-ray spectroscopy (EDX) (Figure 1c). On the other hand, the zeta potential was examined, and it was reported at −25 mv, which indicated the good stability of the synthesized ZnO-NPs.

### 3.2. Proteolytic Enzyme Activity

**Figure 2:**
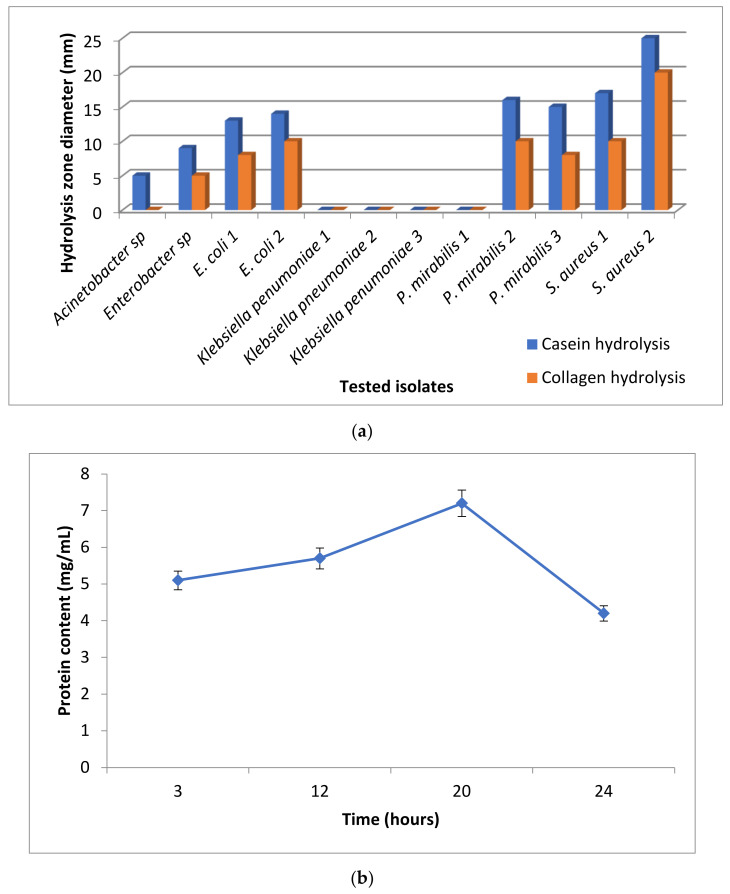
Enzyme activity using the hydrolysis zone method (**a**) and *S. aureus* strain 2 enzyme activity in azocasein substrate (**b**).

The proteolytic activity of the isolated bacteria was assessed on casein and collagen substrates (used as the protein sources). Figure 2a illustrated that the *S. aureus* strain two had a significant proteolytic activity with a hydrolysis zone of a 25 mm and 20 mm diameter in casein and collagen agar plates, respectively. Hence, the *S. aureus* strain two was chosen for further analyses. The enzyme activity was measured by using the Folin–Lowry method at different time intervals (Figure 2b). The highest amount of hydrolyzed protein meant the highest proteolytic activity, which was recorded after 20 h incubation with the azocasein substrate (7.2 mg/mL).

### 3.3. In Vitro Studies

#### 3.3.1. Antibacterial Activity of the Prepared Nanoparticles against the Potent Proteolytic Bacteria

Disc diffusion and microdilution techniques were used to assess the antibacterial activity of the prepared ZnO nanoparticles in vitro. Data revealed that ZnO-NPs had a strong bactericidal activity with an inhibition zone diameter of 23 mm and a minimum inhibitory concentration reaching 8 µg/mL against the proteolytic bacterium (*S. aureus* strain two).

#### 3.3.2. Cytotoxic Effect of the Synthesized ZnO Nanoparticles

**Figure 3:**
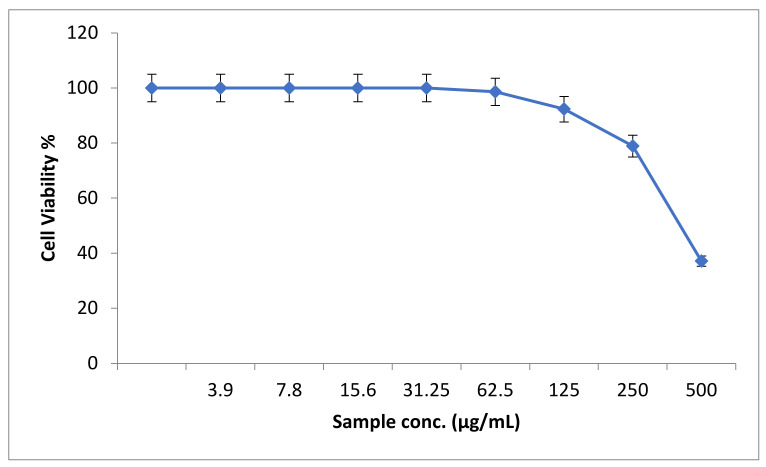
The percentage of normal mouse myoblast cells viability upon treatment with ZnO nanoparticles.

In an attempt to study the in vitro cytotoxic effect of the prepared ZnO nanoparticles, the cell proliferation using mouse myoblasts was tested. It was noticed that, at 500 µg/mL of ZnO-NPs, the mouse myoblast cell viability was 24.8%, while the ZnO-NPs CC50 was 344.16 µg/mL (Figure 3).

### 3.4. In Vivo Study

#### 3.4.1. Histopathological Study

**Figure 4:**
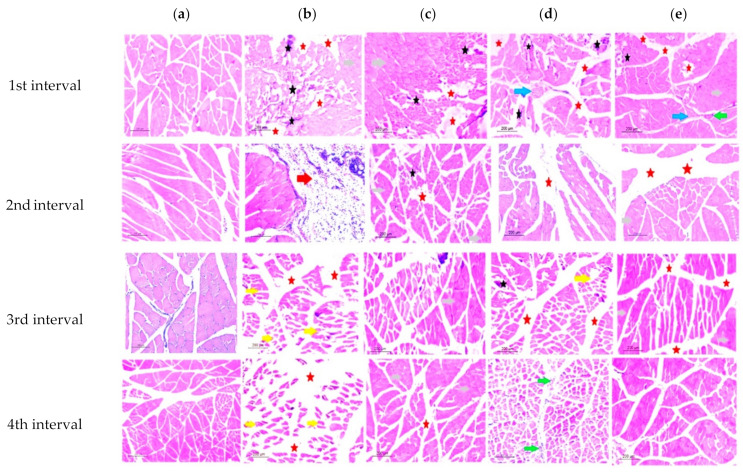
Photomicrographs of transverse section from *biceps femoris*: (**a**) (control group, group I); (**b**) (infected group, group II); (**c**) (ZnO-NP-treated group, group III); (**d**) (physically trained group, group IV); (**e**) (combined treated group, group V). Cellular infiltrate (black stars); necrotic gaps (red stars); nerve bundles (blue arrow); ischemic muscle fibers (grey arrow); dilated blood vessel (green arrow); Edema (red arrow); atrophic skeletal myocytes (yellow arrow). (Scale bars: 200 μm).

The control group’s cross-sectional faces of striated skeletal muscle fibers had polyhedral multisided muscle fibers arranged in groups. Cross-sections of myofibrils in the cytoplasm and the perimysium surrounding the fascicles created by muscle fibers could be seen under the sarcolemma of muscle fibers and in the perimysium surrounding the fascicles formed by muscle fibers (Figure 4a).

In the infected nontreated rat group (group II) muscle cross-sections, multiple neighboring muscle fibers showed some degeneration indices. An early degradation in the first interval was indicated by the increased cellular infiltration along with an observed loss of myofibrils. These symptoms of degeneration progressed across the four intervals, leading to muscular atrophy and pyomyositis in the fourth interval, while, in the second interval, the increasing hydrostatic pressure caused a remarkable muscle edema. Moreover, in the third interval, a significant increase in perimysium connective tissue was observed with many atrophic skeletal myocytes (Figure 4b).

In the ZnO-NP-treated group (group III), ischemic muscle fibers were noticed, causing a significant inflammatory response, with numerous cellular infiltrates and tiny necrotic gaps. These responses stimulated the muscle regeneration through dormant satellite cell activation. Furthermore, despite the persistence of ischemic tissue, the relevance of regeneration myofibers was proven by the progressive recovery of the muscle histoarchitecture, specifically in the fourth interval, as seen by the reduction in the perimysium connective tissue (Figure 4c).

On the other hand, the physiotherapeutic trained group (group IV) displayed cellular infiltration observed in the first interval. Muscle bundles were separated by perimysium connective tissue. The existence of a nerve trunk was seen as an indication of reflex arc activation, demonstrating the impact of the physical activity on muscle regeneration in the second interval. Increased variance in the fiber diameter, dispersed necrotic and regenerating muscle fibers, and an increment in perimysial and/or endomysial connective tissue were major observed signs of nonspecific histological abnormalities (Figure 4d). Moreover, the fourth interval revealed an increased number of capillaries with endothelial hyperplasia and lumen widening of the remaining capillaries, indicating that additional regeneration was occurring (evidenced by inhomogeneous staining).

Over the four intervals, in group V (the combined treatment regimen of ZnO-NPs and the physiotherapeutic program), the least deterioration signs were recorded compared to other groups, indicating a synergistic impact of the nanoparticle treatment and the applied physical exercise. In the first interval, less cellular infiltrate dispersed in the ischemic myocytes in the surrounding connective tissue, with angiogenesis and nerve bundles being observed (Figure 4e). The last interval photomicrographs showed a substantial degree of regenerated myofibers, as shown by the huge, plump, mature, and dystrophic myofibers with central nuclei that seemed to spread uniformly throughout the tissue, as well as the drastic drop in the perimysium observed across the intervals.

#### 3.4.2. Blood Biochemical Analysis

The total WBC was significantly increased by 0.05 values throughout all intervals, with group V having the most significant value at the last interval. The WBC profile displayed the equilibrium between granulocyte production and WBC production. The increased WBC could indicate low-grade inflammation, implying that the chemical–physical treatment group (group V) was nearly regenerated at the last interval, and the inflammation vanished (Table 1 and Table S1). At all intervals, eosinophils had little difference between experimental groups, indicating that these cells play a minor role in bacterial infection. On the one hand, the last interval revealed a substantial difference between groups, particularly in the physically trained group (group IV), which had the highest neutrophil count. Lymphocytes showed an abrupt decrease in the second interval in group IV, which was reversed in the final two intervals to improve the healing process. On the other hand, the infected nontreated group demonstrated the fewest lymphocytes, indicating bacterial dystrophic activity. The monocyte count increased to boost the immunodefense against infectious pathogens and minimize the harmful inflammatory conditions, particularly in the third interval in groups III and V, reflecting the ZnO-NP complicity in monocyte activation.

Furthermore, there was a steady decline in the albumin rate in all experimental groups, with the four intervals revealing no significant hypoalbuminemia, emphasizing the transitory decrease that was restored at the end of the experiment (Table 2 and Table S2). Furthermore, all experimental groups’ SGOT and SGPT levels increased significantly in the blood during the second time interval. Our findings revealed a considerable rise in alkaline phosphatase (ALP) levels, reflecting the infection consequences. Groups II and III had the greatest alkaline phosphatase levels compared to groups IV and V. The physically trained group had a lower ALP value than the other experimental groups and was close to the control group at all time intervals, indicating a higher density of muscular mass and emphasizing the exercise effect on muscles. Creatine kinase levels were lower in all the experimental groups, and group II had the lowest value, but the physically trained group (group IV) had a value that was closer to that of the normal control group (group I). LDH levels increased gradually over the first and second intervals, but decreased significantly during the third and fourth intervals in all the experimental groups, emphasizing the treatment and immunization importance.

#### 3.4.3. Radiological Analysis Results

**Figure 5:**
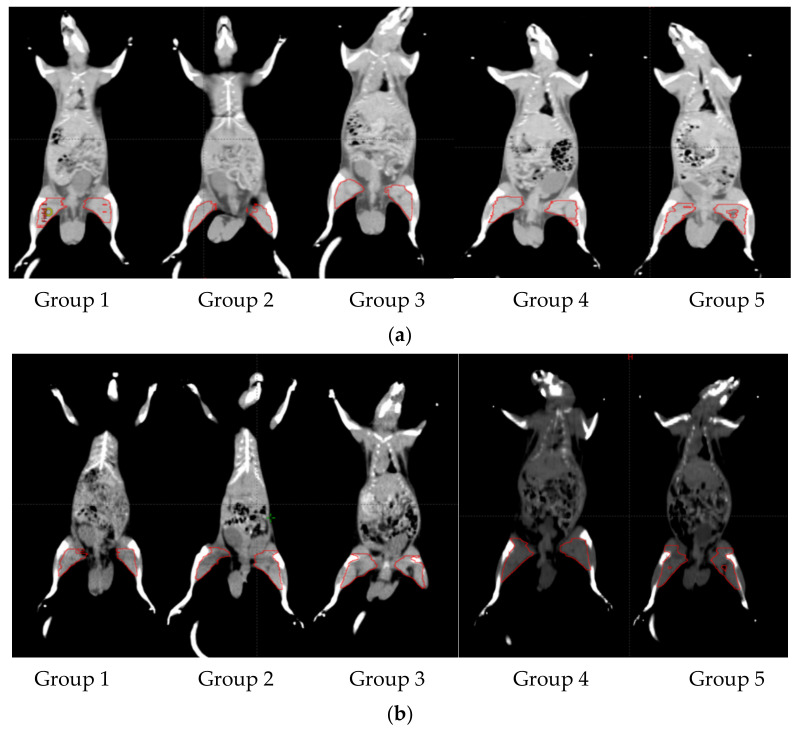
CT muscle scan showing first (**a**) and last intervals (**b**).

The CT graphs quantitatively confirmed the damage degree of the rat *biceps femoris* muscle on the left and right sides (Figure 5). Generally, both models of groups four and five indicated that the increase in muscle size in the damaged muscle was comparable to previous results at all time intervals. Subsequently, in comparison to the previous histological results, group five revealed the best results in myofibers histological features as well as muscle size (Table 3).

### 3.5. Principal Component Analysis

**Figure 6:**
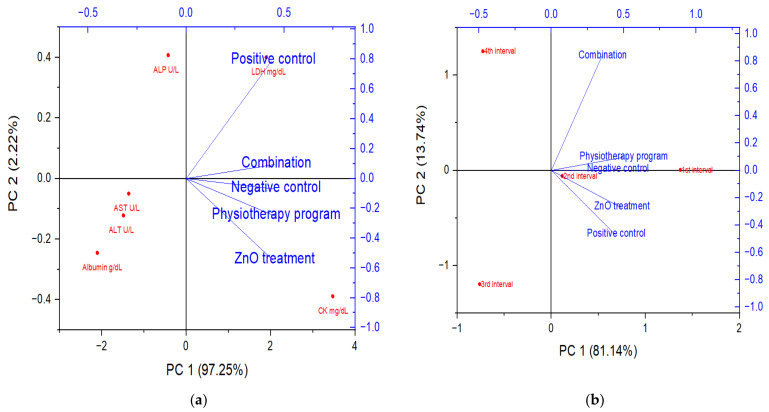
PCA of experimental rat groups where (**a**) the biochemical parameters and (**b**) the muscle size.

The principal component analysis (PCA) was used for the exploration of the collected data for the experimental rat groups regarding the biochemical and muscle size (dependent variables) (Figure 6). The biochemical investigations among the experimental rat groups explained over 97.25% of the total variability (Figure 6a). The first component (PC1) separated between the rat groups where the rat group received the combination protocol was linked and related to the negative (placebo) group as dependent variables. Similar observations were noticed in Figure 6b regarding the rats’ muscle sizes in different groups, with explained total variabilities reaching 81.14%. The observed analysis data revealed that the combination effect between the physiotherapeutic program and the synthesized nanoparticles enhanced the biochemical and physiological parameters, while the physiotherapeutic program alone enhanced the muscle mass specially in the first and second intervals.

## 4. Discussion

One of the most important issues in global health care is the development of antibiotic resistance in microorganisms. Extracellular proteolytic enzymes are bacterial virulence factors that have key roles in host colonization by assisting pathogen spread into host tissues. They also improve the pathogen survival and growth by increasing (a) the availability of amino acids and (b) toxin diffusion [20,21].

The exploitation of demand-satisfying nanoparticles through the development of nanotechnology over the last few decades has led to the widespread usage of nanomaterials in biomedicine and the emergence of the crucial field known as “nanomedicine” [22]. It was established that the internalization process of cells may be considerably impacted by the nanosize of nanomaterials, which would have a considerable impact on their therapeutic efficacy. Nanosized nanomaterials can be readily absorbed by cells, increasing the drug concentration at the site of the lesion, but excessively big (>300 mm) nanomaterials are more likely to be phagocytosed and removed by cells [23]. Pharmaceutical molecules have an extremely small size, a limited bioavailability, and a short half-life, which makes them more susceptible to being scavenged or destroyed by the immune system of the body while in circulation [24]. The acceptable nanoscale size is, therefore, very favorable for the application of therapeutics. Additionally, a crucial component in supplying medications to lesion sites is the interaction between the drug and the biological surroundings [25]. Due to most nanomaterials being easy to modify, they can typically be decorated in a way that maintains a good balance with the physiological environment of the human body, making the most of their possibilities in the medical field. Numerous nanoparticles can also act as delivery systems for medications, shielding them from deterioration to improve therapeutic effectiveness [26]. Numerous investigations have demonstrated that metal nanoparticles and their oxides are one of the most promising strategies for combatting antibiotic resistance in bacteria [27]. Specifically, zinc oxide nanoparticles (ZnO-NPs), which proved to have significant antibacterial activity against a broad spectrum of microbes [28]. Zn^2+^ ions are released in the aqueous suspension when zinc oxide (ZnO) particles partially dissolve, contributing to ZnO’s antibacterial activity [29], dependent on their size [30,31,32]. The higher antibacterial activity can be explained in that the smaller the nanoparticle (NP) size, the easier they can pass through the bacterial nanopores [33]. Although superparamagnetic iron oxide nanoparticles (SPIONs) are the most widely employed functional nanomaterials as antibacterial agents, their medicinal uses are limited by their propensity to aggregate through dipole–dipole interactions between the magnetic particles. According to recent research, titanium dioxide (TiO_2_) and zinc oxide (ZnO) have a selective toxicity to bacterial cells while exerting little impact on human cells. Moreover, Mendes et al. [34] explained that zinc oxide (ZnO), magnesium oxide (MgO), and titanium dioxide (TiO_2_) are considered among the safest pharmaceutical medications. The impact of ZnO as a reactive oxygen species source is an essential key molecule that promotes the reversal of muscle atrophy through the use of activating proteases and decreasing protein synthesis [35]. An animal model mouse with severe pyomyositis caused by biofilm-forming bacteria had a high mortality rate. The communities receiving treatment with these NPs also showed no damage to the healthy tissues and maintained close junctions and muscular structure [36].

On the other hand, exercise can be used to build muscular strength and endurance. Hamm et al. [37] proposed that a threshold could be established to guide appropriate exercise prescription for individuals with muscular atrophy. Kimura et al. [38] demonstrated that immobility might minimize the muscle fiber necrosis in instances with muscular dystrophy. Antiapoptotic drugs, as well as low-level laser therapy (LLLT), may be potential treatments for acute atrophy by inhibiting the clearance of myonuclei [39]. A systematic review of LLLT with single-wavelength laser probes (1: 904 nm vs. control, and 2: 940 nm vs. 808 nm vs. 658 nm) was conducted. It was found that a significant difference was noticed with 658 nm, but no supportive evidence was found for laser probes using other wavelengths in the treatment process of pressure ulcers [40]. Lopes-Martins et al. [41] evaluated the LLLT effects in an experimental model of electrical nerve stimulation used to create tiredness in rats via tetanic tibial muscular contractions to assess the possible decline in muscle strength with respect to the increment in CK levels. To the best of our knowledge, only a small amount of research has investigated the relationship between CK levels and muscle damage [42]. The importance of increased blood CK levels following physical activity was reported in relation to the degree of muscle cell injury or disruption [43].

The purpose of this study was to (a) investigate the antibacterial activity of zinc oxide nanoparticles applied as a treatment to the induced pyomyositis of the *biceps femoris* in rats and (b) evaluate if swimming and LLLT as a physiotherapeutic regimen could reduce the negative effects of pyomyositis. The findings of our study were supported with the findings of previous studies indicating that LLLT could reduce CK levels. In an experimental animal model of Achilles tendon damage, Fillipin et al. [44] found that LLLT decreased histological abnormalities, collagen concentration, and oxidative stress. Smerdu and Perše [45] discovered that mice subjected to swimming had fewer 2B fibers and more 2D fibers in the extensor *digitorum longus* muscle. Because the *biceps femoris* and extensor *digitorum longus* muscles perform identical tasks in the femur of mice, the difference could only be explained by using LLLT as complementary to the physiotherapeutic protocol in the current study. Swimming, a mechanotransduction mechanism, can prevent muscle atrophy and enhance protein synthesis [46]. Cells respond to the mechanical stimuli by converting them to biochemical signals that elicit a specific cellular response [47]; this mechanical stimulus is transmitted to the extracellular matrix components, which are primarily composed of collagens and glycoproteins that surround the muscle fibers.

The reduction in the myofiber diameter is the characteristic histological sign of skeletal muscle atrophy and pyomyositis [48], as seen in group II. Myofibers that are affected are often smaller, rounded or angular, and have hypereosinophilic sarcoplasm. Degenerated, necrotic, or hyalinized myofibers, split or fractured myofibers, and myofibers with central nuclei are all possible histopathological characteristics.

Histopathology results of the present study were supported by Nagy et al. [49], who observed that the myofiber regeneration and the number of capillary increments in the ischemic hind limb emerged in our ZnO-NP-treated group, in comparison to the combination treated group (group V) with improved muscle histoarchitecture, demonstrating the significant impact of the physiotherapeutic program.

It was proposed that an increased WBC is attributable to increased lymphopoiesis and/or the accelerated release of lymphocytes from lymph myeloid tissue [50]. According to Kwon et al. [51], sarcopenia is highly connected to platelets and WBC, which are commonly employed as inflammatory biomarkers. Neutrophils are known to have three primary antimicrobial functions: phagocytosis, degranulation, and the release of nuclear material in the form of neutrophil extracellular traps (NETs) [52]. According to our findings, the increased neutrophils count in all experimental groups, particularly the physically trained group (group IV), indicated the significance of exercise in boosting body immunity to combat illness.

Chronic liver disorders and muscular injuries led to increasing SGPT levels [53]. This might explain why the measured values were greater in the first two intervals, demonstrating the prevalence of muscular atrophy followed by recovery stratification. Furthermore, in certain animal and human trials, reducing chronic, low-grade inflammation using nonsteroidal anti-inflammatory medicines has been shown to reduce ALP levels, causing a protective impact against the muscle mass and function loss [54]. Lactate dehydrogenase (LDH) is a widely distributed enzyme in cells of diverse living systems, catalyzing the interconversion of lactate and pyruvate using the NAD^+^/NADH coenzyme system. Skeletal muscle diseases usually cause increased LDH levels [55]. Our results of group III revealed the anti-inflammatory properties of the prepared ZnO-NPs.

In regard to another key factor determined with computed tomography (CT) imaging, this work studied the morphological alterations and quantitatively assessed the degree of damage in rat femur muscles. The lean body mass assessment via CT scan is already used in cancer patients to detect muscle wasting, and it is more sensitive than changes in the body mass index or waistline [56]. As a result, tracking muscle loss with in vivo imaging technology offers a unique chance to assess when it becomes a life-threatening illness. The loss of muscle mass during *biceps femoris* pyomyositis in group III was related to a significant drop in the mean fiber diameter, as well as the decrease in the number of sarcomeres, as explained by Sachdeva et al. [57].

## 5. Conclusions

It could be concluded that the remarkable induced outcomes of physiotherapeutic programs combined with the ZnO-NP treatment of *biceps femoris* pyomyositis in a rat model may be a ray of hope in the field of muscle degeneration recovery, highlighting the importance and synergism between chemical and physical treatments.

## Figures and Tables

**Table 1:** Hematological parameters of the experimental rat groups.

Hematological Parameters	Negative Control				Physiotherapeutic Program				Combination			
	Week 1	Week 2	Week 3	Week 4	Week 1	Week 2	Week 3	Week 4	Week 1	Week 2	Week 3	Week 4
WBCs	8700 ± 669.2	7200 ± 480.0	8300 ± 592.9	8800 ± 586.7	5500 ± 343.8	10,400 ± 742.9	10,200 ± 680.0	10,350 ± 646.9	4400 ± 366.7	6500 ± 500.0	7000 ± 583.3	12,800 ± 752.9
Eosinophile	3 ± 0.30	2 ± 0.29	3 ± 0.43	2 ± 0.20	4 ± 0.50	4 ± 0.33	4 ± 0.40	3 ± 0.25	3 ± 0.25	2 ± 0.29	2 ± 0.18	2 ± 0.22
Neutrophile	40 ± 3.08	44 ± 4.00	45 ± 4.50	32 ± 3.56	41 ± 3.15	57 ± 5.70	58 ± 5.80	60 ± 5.00	41 ± 3.15	40 ± 4.00	41 ± 4.10	36 ± 2.77
Lymphocyte	52 ± 4.73	51 ± 5.10	56 ± 4.67	62 ± 4.77	52 ± 5.20	37 ± 2.85	55 ± 4.58	59 ± 4.92	52 ± 4.00	58 ± 4.83	58 ± 4.14	59 ± 4.92
Monocyte	5 ± 0.45	3 ± 0.27	5 ± 0.50	4 ± 0.44	3 ± 0.33	2 ± 0.22	3 ± 0.33	3 ± 0.33	4 ± 0.36	3 ± 0.27	3 ± 0.33	3 ± 0.30

**Table 2:** Biochemical parameters of the experimental rat groups.

Biochemical Parameters	Negative Control				Physiotherapeutic Program				Combination			
	Week 1	Week 2	Week 3	Week 4	Week 1	Week 2	Week 3	Week 4	Week 1	Week 2	Week 3	Week 4
LDH mg/dL	1295 ± 86.3	1467 ± 97.8	417 ± 27.8	700 ± 53.8	894 ± 74.5	1091 ± 99.2	584 ± 41.7	540 ± 45.0	558 ± 39.9	1647 ± 137.3	221 ± 18.4	260 ± 21.7
Albumin g/dL	4.2 ± 0.47	3.6 ± 0.30	2.3 ± 0.23	3.8 ± 0.42	3.3 ± 0.37	1.7 ± 0.17	2 ± 0.22	3.5 ± 0.32	3.8 ± 0.35	3.8 ± 0.35	3.3 ± 0.33	3.6 ± 0.36
CK mg/dL	1395 ± 99.6	1070 ± 89.2	1170 ± 90.0	1389 ± 81.7	1507 ± 115.9	1312 ± 93.7	970 ± 88.2	960 ± 68.6	1828 ± 140.6	586 ± 45.1	506 ± 36.1	628 ± 44.9
ALT U/L	99 ± 6.6	237 ± 21.5	77 ± 5.9	104 ± 9.5	100 ± 7.7	111 ± 7.9	112 ± 8.0	115 ± 9.6	51 ± 3.9	262 ± 23.8	95 ± 6.3	56 ± 3.7
AST U/L	123 ± 7.7	140 ± 8.2	135 ± 10.4	77 ± 5.9	140 ± 10.0	171 ± 15.5	172 ± 11.5	175 ± 13.5	110 ± 9.2	219 ± 12.9	91 ± 5.7	81 ± 6.2
ALP U/L	319 ± 26.6	261 ± 16.3	281 ± 18.7	153 ± 13.9	265 ± 24.1	311 ± 19.4	240 ± 16.0	255 ± 15.0	235 ± 13.8	386 ± 27.6	199 ± 18.1	298 ± 21.3

**Table 3:** Different studied groups regarding the muscle size at different time intervals.

Time Intervals	Group 1	Group 2	Group 3	Group 4	Group 5
First interval	6.7	6.5	6.9	6.7	6.4
Second interval	6.5	5.9	6.5	6.1	6.3
Third interval	6.5	5.1	6.2	5.7	6
Fourth interval	6.5	4.2	6	5.8	6.3

## Data Availability

Not applicable.

## Supplementary Materials

The following supporting information can be downloaded at: https://www.mdpi.com/article/10.3390/biology11101393/s1, Table S1: hematological parameters of the experimental rat groups, Table S2: biochemical parameters of the experimental rat groups.
