# Supplementary material for: Physiotherapeutic Protocol and ZnO Nanoparticles: A Combined Novel Treatment Program against Bacterial Pyomyositis
Source: Biology (Basel). 2022 Sep 23;11(10):1393. doi: 10.3390/biology11101393 (PMC9598154; doi:10.3390/biology11101393)
Supplement: Supplementary file 1 [file biology-11-01393-s001.zip › biology-1916650-supplementary.pdf]

biology

Supplementary Material

Physiotherapeutic Protocol and ZnO Nanoparticles: A Combined Novel Treatment Program against Bacterial Pyomyositis

Hesham El-Shaer, Bassma H. Elwakil, Basant A. Bakr, Ahmed M. Eldrieny, Mostafa El-Khatib, KhimPhin Chong and Amr A. Abo Gazia

Table S1. Hematological parameters of the experimental rat groups.

Hematological Parameters	Positive control				ZnO treatment			
	Week 1	Week 2	Week 3	Week 4	Week 1	Week 2	Week 3	Week 4
WBCs	8100±623.1	7500±535.7	7900±493.8	7700±481.3	5700±356.3	7800±487.5	8500±607.1	8700±621.4
Eosinophile	4±0.40	3±0.38	1±0.13	3±0.38	2±0.18	2±0.17	3±0.30	2±0.29
Neutrophile	30±3.00	38±3.80	38±4.22	38±2.92	35±2.92	40±3.33	40±4.00	33±3.67
Lymphocyte	61±4.69	56±4.31	37±2.85	55±3.93	60±6.00	55±4.23	57±4.75	62±6.20
Monocyte	5±0.50	3±0.30	2±0.18	4±0.40	3±0.33	3±0.33	4±0.40	3±0.33

Table S2. Biochemical parameters of the experimental rat groups.

Biochemical Parameters	Positive control				ZnO treatment			
	Week 1	Week 2	Week 3	Week 4	Week 1	Week 2	Week 3	Week 4
LDH mg/dL	686±42.9	753±50.2	301±18.8	271±15.9	688±40.5	937±72.1	646±46.1	963±64.2
Albumin g/dL	3.7±0.34	2.5±0.23	3±0.30	4.3±0.48	4.3±0.43	3.3±0.37	4.1±0.34	4.3±0. 43
CK mg/dL	791±60.8	191±12.7	716±47.7	472±33.7	1904±146.5	1330±88.7	1280±85.3	1924±128.3
ALT U/L	65±5.9	140±9.3	91±5.7	54±3.9	152±10.1	274±18.3	73±5.2	70±5.4
AST U/L	108±7.2	185±10.9	112±10.2	69±4.3	109±6.4	205±12.1	167±13.9	133±11.1
ALP U/L	317±24.4	427±28.5	266±24.2	236±21.5	341±31.0	528±31.1	253±16.9	303±20.2
